# How School‐Based Interventions Shape the Developing Brain during 3‐12 Years: A Systematic Review and Exploratory Meta‐analysis

**DOI:** 10.1016/j.dcn.2026.101831

**Published:** 2026-10-01

**Authors:** Jingwen Li, Runke Huang, Hui Li

**Affiliations:** a School of Humanities and Social Science, The Chinese University of Hong Kong - Shenzhen, Shenzhen, Guangdong, 518172, PR China; b Faculty of Education and Human Development, The Education University of Hong Kong, Hong Kong Special Administrative Region of China; c Macquarie School of Education, Macquarie University, NSW, Australia

**Keywords:** School-based intervention, Educational intervention, Neuroimaging, Neurodevelopment, Cognitive and emotional development

## Abstract

The effects of school-based interventions (SBIs) on children’s neurodevelopment are poorly synthesized. This systematic review integrates neuroscientific evidence on how structured educational activities influence brain development in children aged 3–12 years. Following PRISMA guidelines, we identified 17 empirical studies (published 2014–2025) using functional near-infrared spectroscopy (fNIRS), electroencephalography (EEG), magnetic resonance imaging (MRI), or magnetoencephalography (MEG). Of these, 8 studies were included in a random-effects meta-analysis and all 17 in a thematic synthesis. The meta-analysis revealed a preliminary association between SBIs and reduced amygdala activity during stress regulation tasks (*r* = –.28, 95% CI [–.50, –.02]), based on two studies, which should be interpreted as exploratory rather than confirmatory. No significant pooled effect for executive function, likely due to high heterogeneity. Thematic analysis distinguishes between mechanisms of plasticity and markers of intervention effectiveness: the first three characterize intra-individual mechanisms of experience-dependent plasticity, including (1) executive function interventions engaged prefrontal networks through both increased recruitment and enhanced neural efficiency; (2) socio-emotional programs modulated limbic-prefrontal circuits; (3) language and auditory programs shaped sensory-specific electrophysiological markers and cortical specialization; and the fourth theme identifies a neural marker of the effective process of intervention delivery, demonstrating that (4) teacher-child inter-brain synchrony was positively correlated with learning outcomes. By integrating findings across neural, cognitive, and affective domains, this review provides a conceptual framework linking pedagogical practices to neural plasticity and advances the understanding of the neurofunctional pathways through which educational experiences shape the developing brain.

## Highlights

• SBIs were linked to reduced amygdala activity, improving stress regulation.
• EF-focused SBIs engaged prefrontal networks through upregulation and efficiency.
• Language- and auditory SBIs enhanced MMN/P3a responses and cortical specialization.
• Teacher–child IFG synchrony correlated positively with learning outcomes.

## Graphical Abstract

**Figure:**
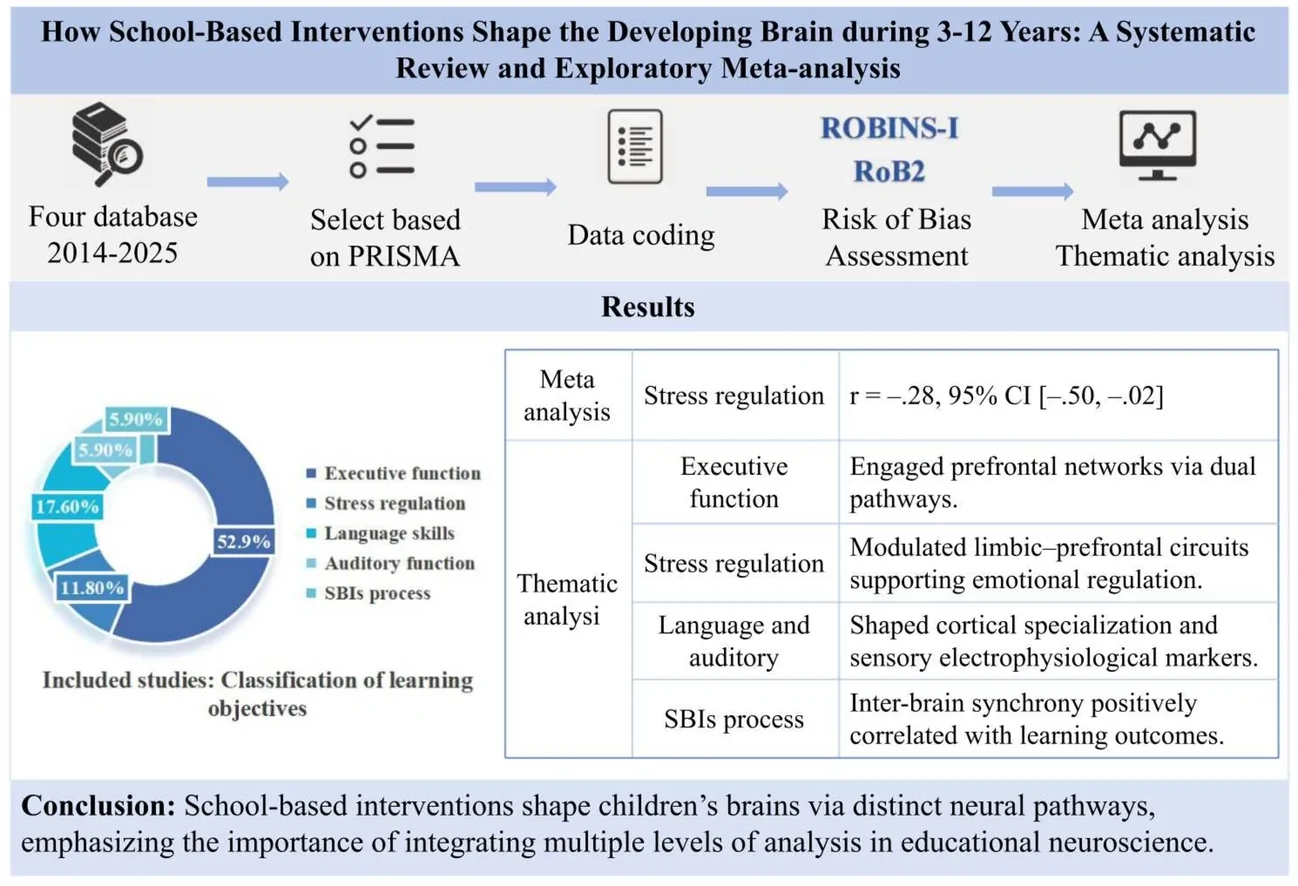

## 1 Introduction

School-based interventions (SBIs) are typically defined as structured, curriculum-aligned, and teacher-guided programs implemented to enhance students’ emotional, behavioral, or social functioning (Durlak et al., 2011, Frank et al., 2021). While prior research has primarily examined SBIs within formal schooling—focusing on primary or secondary education—this review adopts a broader developmental perspective that includes children aged 3–12 years, spanning both early and middle childhood. The 3–12 age range represents a critical period of neural plasticity, during which brain systems supporting language, executive control, and emotion regulation undergo rapid reorganization (Knudsen, 2004, White et al., 2017). Consequently, the structured experiences provided by SBIs are uniquely positioned to shape the neural circuits underlying learning and behavior. A growing body of neuroimaging research has begun to illuminate these effects; for example, studies have linked dialogic reading to enhanced language-related brain function (Romeo et al., 2018), physical activity to modulated executive control networks (Chaddock-Heyman et al., 2013), and mindfulness to altered amygdala–prefrontal connectivity associated with emotion regulation (Bauer et al., 2019).

Despite these promising individual findings, a coherent understanding of the neurodevelopmental impact of SBIs remains elusive. The current literature is highly fragmented, hindering the translation of neuroscientific insights into evidence-based educational practice. This fragmentation manifests in three primary ways. First, interventions are exceptionally diverse, ranging from specific academic instruction (Ding et al., 2024) to broader wellness activities like music or outdoor education (Chobert et al., 2014, Dettweiler et al., 2022), making it difficult to compare their neural signatures. Second, studies employ different neuroimaging modalities (e.g., fMRI, fNIRS, EEG) that capture distinct aspects of brain function, from hemodynamic responses to electrophysiological activity, complicating direct synthesis. Third, and most critically, even interventions targeting the same developmental outcome report inconsistent and sometimes opposing neural effects. For instance, some studies report increased prefrontal activation following an executive function intervention, interpreted as greater neural recruitment, while others report decreased activation, interpreted as enhanced neural efficiency (Soltanlou et al., 2018, Zhong et al., 2024b). Without a systematic synthesis, it is unclear whether there are consistent patterns of neural change or how different interventions achieve similar behavioral goals via distinct neural pathways.

The present study was designed to address these gaps by systematically integrating neuroimaging evidence on SBIs in children aged 3–12 years. This review has two primary aims. First, we conduct an exploratory meta-analysis to provide a preliminary estimate of the overall strength of association between SBIs and neurofunctional outcomes in key domains. Second, we perform a qualitative thematic synthesis to identify and characterize the diverse neural mechanisms through which different SBIs produce their effects. By combining quantitative and qualitative approaches, this review seeks to move beyond isolated findings to build a more cohesive conceptual framework. In doing so, it aims to bridge the gap between educational practice and developmental neuroscience, providing a more comprehensive account of how structured learning experiences shape the developing brain.

### 1.1 Heterogeneity in the neurofunctional outcomes of school-based interventions

A growing body of neuroimaging research seeks to understand how school-based interventions (SBIs) shape the developing brain. These interventions span a range of educational practices that foster children’s academic, emotional, and social development within structured school environments (Cai et al., 2025, Frank et al., 2021). By leveraging the heightened neural plasticity of childhood (Knudsen, 2004), SBIs offer a powerful real-world model for examining experience-dependent changes in the brain’s functional and structural architecture.

A central challenge in this literature is the striking heterogeneity of neural outcomes, even for interventions within the same domain. In academic learning, for example, SBIs can produce opposing patterns of brain activation. Some studies on mathematics tutoring report enhanced fronto-parietal connectivity, suggesting the strengthening of networks for working memory and numerical cognition (Supekar et al., 2013). Conversely, other studies find reduced activation in parietal regions following arithmetic training, a change interpreted as a gain in neural efficiency, where the brain accomplishes the task with fewer resources (Soltanlou et al., 2018). A similar pattern appears in reading research, where intensive phonics instruction has been linked to increased activation in left-hemisphere language and visual regions (Shaywitz et al., 2004), while other paradigms report decreases. These divergent findings illustrate that SBIs can influence neural function through distinct pathways of either increased network recruitment or enhanced processing efficiency, complicating efforts to define a single “signature” of a successful intervention.

This inconsistency extends beyond academic subjects to socio-emotional and enrichment programs. Mindfulness-based SEL programs, for instance, have been shown to strengthen prefrontal-limbic regulation, resulting in reduced physiological stress and improved prosocial behavior (Schonert-Reichl et al., 2015), aligning with behavioral evidence that SEL can bolster self-regulatory skills (Durlak et al., 2011). However, the evidence is not uniformly consistent, particularly for non-academic activities. While some randomized trials show that music training enhances auditory cortical processing and cross-hemispheric connectivity (Habibi et al., 2017, Hyde et al., 2009), other large-scale studies report no such association. Likewise, while aerobic exercise has been associated with greater hippocampal volume and modulated executive networks (Chaddock-Heyman et al., 2013, Tang et al., 2012), other rigorous trials have reported null effects on white matter microstructure even when behavioral improvements were observed (Rodriguez-Ayllon et al., 2024). This potential dissociation between behavioral and neural outcomes suggests that the benefits of such interventions are not universal and may depend on the specific mechanisms engaged.

Taken together, the existing literature demonstrates that while SBIs can influence neurodevelopment, the findings are highly variable across studies. This inconsistency reflects the vast diversity in intervention designs, participant characteristics (e.g., age, developmental stage), task paradigms, and neuroimaging methodologies (van der Zwaag et al., 2009). This landscape of disparate findings underscores an urgent need for a systematic synthesis to determine whether reliable patterns exist and to build a more coherent evidence base regarding the effects of SBIs on children’s neural functioning.

### 1.2 Multiple neural pathways underlying intervention effects

The capacity for SBIs to alter brain function is rooted in the principle of neural plasticity, whereby repeated, meaningful experiences reorganize neural circuits to support new skills (Greenough et al., 1987). This experience-dependent shaping typically follows the principle of “domain-specificity”, where different forms of intervention are expected to engage distinct neural systems specialized for those functions (Johnson, 2011). However, the relationship between an intervention and its neural outcome is often more complex than a simple one-to-one mapping. Different interventions may achieve similar behavioral improvements through markedly different neural pathways—a phenomenon known as “multiple neural realizability” (Figdor, 2010). From this perspective, understanding how SBIs shape the developing brain requires not only identifying their domain-specific targets, but also tracing the multiple neural routes through which similar functional outcomes may be achieved.

Evidence for this domain-specificity is well-documented. Academic interventions primarily reshape cortical networks dedicated to symbolic processing. For instance, literacy instruction strengthens temporo-parietal circuits for grapheme-phoneme mapping and the occipito-temporal “visual word form area” for fluent word recognition (Dehaene and Cohen, 2011, Shaywitz et al., 2004). Mathematics training, in contrast, engages the intraparietal sulcus for numerical magnitude processing and prefrontal networks for working memory and strategy use (Istomina and Arsalidou, 2024, Supekar et al., 2013). Similarly, interventions targeting socio-emotional skills, such as social-emotional learning (SEL) programs, primarily modulate prefrontal-limbic circuitry, reflecting strengthened top-down regulation of emotion (Luo and Yu, 2015, Schonert-Reichl et al., 2015). Even enrichment activities like arts or physical training produce specific effects, such as modifying white matter connectivity or enhancing hippocampal function (Chaddock-Heyman et al., 2013, Habibi et al., 2017).

The presence of multiple neural realizability (Price and Friston, 2005) suggests that interventions with similar behavioral goals can operate through distinct, and sometimes opposing, neural mechanisms. This demonstrates that there are often multiple pathways to a common developmental benefit. For example, interventions designed to improve emotion regulation can work differently: mindfulness-based practices tend to strengthen top-down control from the prefrontal and anterior cingulate cortices (Schonert-Reichl et al., 2015), whereas other SEL approaches may focus on modulating bottom-up reactivity in the amygdala (Luo and Yu, 2015). Likewise, both STEM learning and physical activity can enhance executive functions, but they appear to do so via different routes. STEM practice has been associated with greater neural efficiency (i.e., reduced activation) in prefrontal regions, while physical exercise may increase cerebral blood flow and neural recruitment in attention-related areas (Ding et al., 2024, Zhong et al., 2024b).

This complexity highlights a critical gap in the literature. The existence of multiple neural pathways means that a purely quantitative synthesis—which aggregates data toward a single mean effect—may obscure functionally important distinctions or misinterpret heterogeneity as simple error. Therefore, this review will complement its quantitative analysis with a qualitative thematic synthesis. This integrative approach is essential for capturing the nuanced patterns of neural plasticity across different interventions and for developing a deeper understanding of how structured learning experiences shape the developing brain.

### 1.3 The present study

A systematic integration of neuroimaging findings is essential for bridging the gap between educational practice and developmental neuroscience, creating an evidence base for designing effective, brain-informed interventions (Fox et al., 2010). While previous reviews have provided valuable insights, their scope has often been limited, leaving a fragmented understanding of the neural effects of SBIs. Existing syntheses are often constrained in three key ways. First, most focus on a single domain, such as mindfulness (Tang et al., 2012), reading (Perdue et al., 2022), or music (Habibi et al., 2017), preventing a cross-domain understanding of how different types of learning shape the brain. Second, their conclusions are frequently based on narrow age ranges (e.g., adolescents), leaving the effects on younger children in early and middle childhood systematically underexplored. Third, even within a single domain, high methodological heterogeneity often leads to mixed or non-significant findings, highlighting the challenge of quantitative synthesis alone (Perdue et al., 2022). To date, no review has systematically integrated neural mechanisms across cognitive, linguistic, and socio-emotional domains for children aged 3–12.

The present systematic review and exploratory meta-analysis was designed to address these limitations by synthesizing empirical neuroimaging studies (MRI, fNIRS, EEG, MEG) published between January 2014 and May 2025. By focusing on the critical developmental period of 3–12 years, we aim to provide a comprehensive and age-sensitive overview of the field. Specifically, we pursue two primary objectives guided by the following research questions:

1. What is the strength of association between SBIs and children’s neurofunctional outcomes? To address this, we conduct an exploratory meta-analysis to quantitatively estimate the overall effect sizes in key developmental domains (e.g., executive function, stress regulation).

2. What neural mechanisms underlie the effects of different SBIs? We employ a qualitative thematic synthesis to identify and characterize the specific and potentially convergent neural pathways through which interventions targeting similar goals (e.g., improved self-regulation) exert their influence.

By combining quantitative and qualitative approaches, this review aims to consolidate disparate findings, clarify the diverse ways in which learning experiences shape neural plasticity, and provide a more integrated framework for future research and practice.

## 2 Methods

This review systematically synthesized recent empirical evidence on how SBIs shape neural development in children. Specifically, it aims to evaluate the associations between SBIs and neural outcomes across key developmental domains and to explore the underlying neural mechanisms. The review strictly followed the *Preferred Reporting Items for Systematic Reviews and Meta-Analyses* extension for Systematic Review for literature retrieval and screening (PRISMA; Page et al., 2021). PRISMA is a practical guidance tool for researchers interested in reporting systematic evaluations and is key to achieving objective and effective systematic reviews. Building upon the research questions, the development of this review comprises the following five steps: (1) conducting a comprehensive literature search; (2) applying inclusion and exclusion criteria for study selection; (3) extracting and coding relevant data; (4) assessing study quality and risk of bias using the RoB 2 and ROBINS-ⅠV2; (5) performing quantitative synthesis (meta-analysis) and qualitative thematic synthesis.

### 2.1 Search Strategy

This systematic review used a total of four databases for retrieval, including Web of Science (https://access.clarivate.com/wos/), PubMed (https://pubmed.ncbi.nlm.nih.gov), Scopus (https://www.scopus.com/), and EBSCO (https://www.ebsco.com/). The search covered publications from January 2014 to May 2025 and was completed in June 2025. The 2014 start date was selected to capture a period marked by a rapid increase in empirical research in this area. This growth coincides with the maturation and wider adoption of portable neuroimaging techniques, such as fNIRS and portable EEG, which have enabled a shift from laboratory-based studies to more ecologically valid research conducted in real-world classroom settings (Pinti et al., 2018, Vanderwert and Nelson, 2014).

The search strategy combined Boolean operators (AND, OR) to link four conceptual categories: (1) teaching and interaction terms; (2) intervention scenarios (3) neuroimaging terms; and (4) participant/educational level terms. Combine the four sets of keywords in detail to form the search keywords: (educat* OR learn* OR curriculum OR teach* OR interaction OR instruction OR intervention) AND (school-based OR class-based OR educational) AND (brain OR neuro OR neural OR hyper* OR synchronous) AND (student OR preschooler OR children OR teacher OR primary school OR kindergarten OR preschool). No language restrictions were initially applied; however, only peer-reviewed journal articles available in English were included at the screening stage. Reference lists of included studies were also manually searched to identify additional relevant publications.

### 2.2 Study selection

Specific inclusion and exclusion criteria were established to ensure the relevance and methodological quality of studies in this review. Studies were included if they met all of the following conditions: (1) Empirical research (experimental or observational) with primary data collection; (2) Published in peer-reviewed journals; (3) Written in English; (4) Participants were typical children aged 3–12 years (for dyadic or hyperscanning paradigms, adults were permitted only as instructors and were not included as primary analytical targets); (5) Employed at least one neuroimaging method (e.g., fNIRS, EEG, fMRI, MEG); (6) Published between January 2014 and May 2025; (7) Involved learning activities conducted through SBIs; (8) Reported neural outcomes reflecting brain development or functional changes during or following intervention. A detailed summary of the inclusion and exclusion criteria is presented in Table 1.

**Table 1:** Inclusion/exclusion criteria.

**Criterion**	**Inclusion**	**Exclusion**
**Scope of research**	Empirical studies	Non-empirical studies (reviews, theoretical studies, editorials et al.) Secondary data analysis
**Type of documents**	Peer-reviewed scholarly journal articles	Non-peer-reviewed scholarly journal articles
**Language**	English	Other languages than English
**Participant**	Healthy population students aged 3–12 years old (adults permitted only as instructors in dyadic paradigms)	Special populations (with disabilities, mental developmental disorders, etc.) Less than 3 years old or over 12 years old (except for instructor)
**Method**	Neuroimaging technology multimodal technologies including neuroimaging	Non-neuroimaging technology
**Period**	January, 2014 - May, 2025	Before 2014 and after May 2025
**Article content**	Describe of intervention plan Present research results	No research results available Unable to find original text No entire intervention plan No teacher-student interaction
**Research topic**	Effect of SBIs on teacher or student’s brain function or structure	Articles that not focus on the effect of SBIs on brain

Following the search procedure, a total of 10,887 records were identified across the four databases. After removing 3054 duplicates, 7833 unique records remained for screening. Titles and abstracts were first reviewed against the predefined inclusion and exclusion criteria, resulting in the exclusion of 7573 records that were clearly unrelated to the topic of SBIs and neural development. The remaining records underwent full-text screening. At this stage, 5 articles were excluded because the full text could not be retrieved; 34 articles were excluded due to participants belonging to a special group and not meeting the eligibility criteria; 17 articles were excluded due to participants being over 12 years old; 37 articles were excluded because they did not use brain imaging techniques; 38 articles were excluded due to the lack of a clear teaching interaction plan; 1 article has no research results and has been excluded; 10 articles are excluded as they do not belong to empirical research; and 101 articles were removed because the instructional methods described were not based on school-based intervention. After all screening stages, 17 studies met the inclusion criteria and were retained for final analysis. The detailed process of study identification, screening, eligibility assessment, and inclusion is illustrated in the PRISMA flow diagram (Fig. 1).

**Fig. 1:**
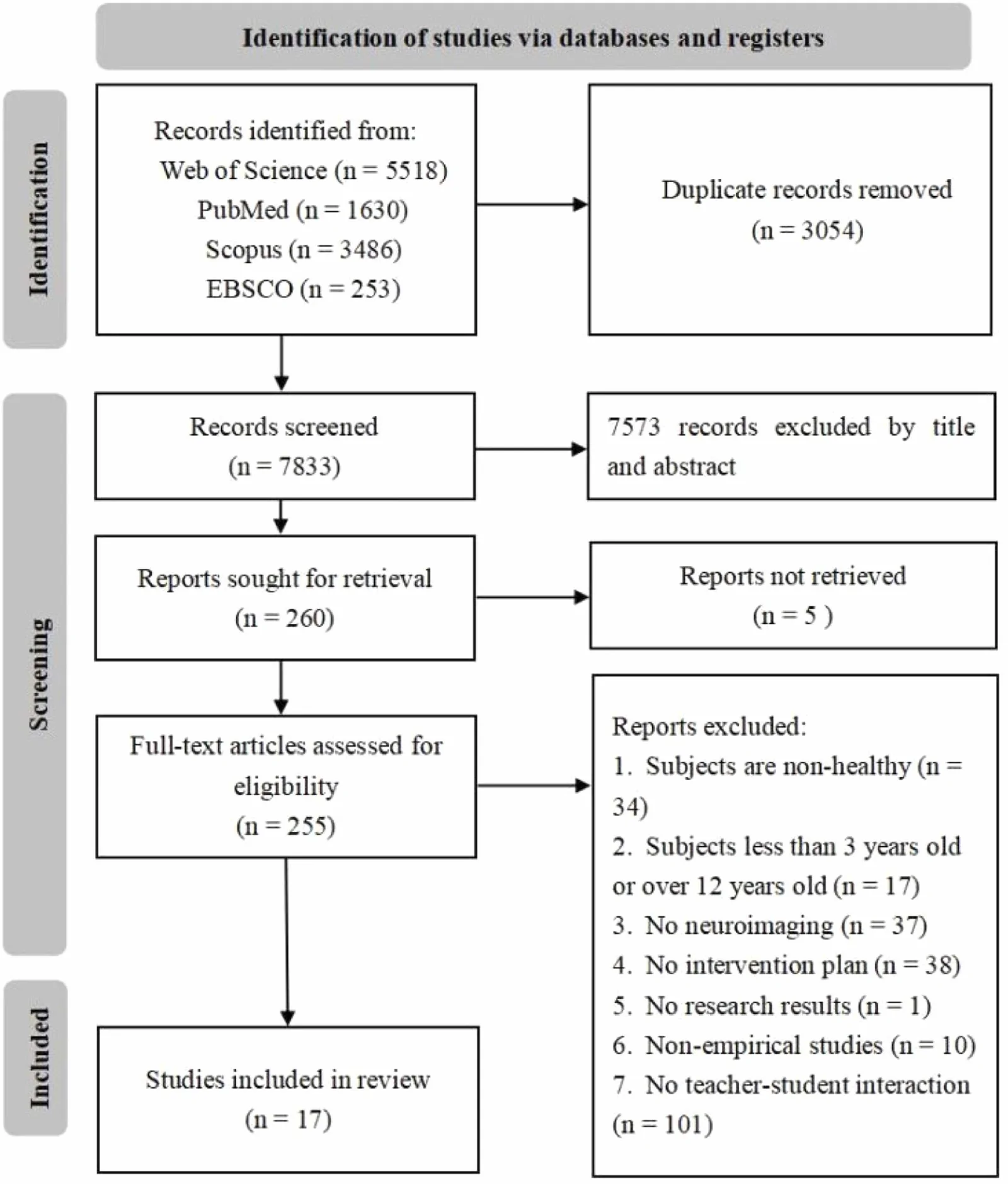
PRISMA flow diagram of literature search and data charting.

### 2.3 Data coding

This systematic review ultimately included 17 studies for coding and analysis. The following information was extracted from these articles as encoding features: author, publication year, country, participant information (sample size, gender, age, and characteristics), methods (neuroimaging techniques, brain regions, intervention content, experimental type, ability to intervene, intervention duration), active/passive learning, neuroimaging results, and relevant analysis results of neuroimaging.

To ensure rigor and impartiality in the coding process, three psychology and education professionals independently coded the studies for this systematic review. One coder was responsible for coding 17 articles, while two specialist coders independently coded neuroimaging-related variables according to their methodological expertise (one for fNIRS and MRI studies, and one for EEG and MEG studies). Before the coding, three coders held a meeting to determine the standards and obtained two coding cases. After independently coding 3 articles each, coders reconvened to examine inter-coder consistency, resolving discrepancies before proceeding with the remaining studies. Divergent items were discussed until full consensus was achieved. Inter-rater reliability, calculated using Cohen’s Kappa coefficient, indicated substantial agreement (*k* = 0.845). The final coding protocol was refined through three iterative rounds of consensus-building.

### 2.4 Study risk of bias assessment

Risk of bias was assessed using appropriate tools for methodological consistency. Fifteen randomized controlled trials were evaluated with RoB 2 (Sterne et al., 2019), and two non-randomized studies with ROBINS-I V2 (Sterne et al., 2016).

RoB 2 evaluates bias across five domains using a three-level rating system (“low”, “some concerns”, or “high”), and determines the overall rating using the “worst domain” approach. Assessments utilized the official Excel tool (https://methods.cochrane.org/bias/resources/rob-2-revised-cochrane-risk-bias-tool-randomized-trials) Similarly, ROBINS-I V2 examines seven domains of potential bias and applies the “worst domain” principle to produce overall ratings of “low”, “moderate”, “serious”, or “critical” risk. The assessments were performed using the ROBINS-I V2 Excel template, independently completed by two trained raters. Inter-rater agreement was verified through Kappa statistics (RoB 2: *k* = 0.733; ROBINS-I V2: *k* = 0.857). Risk-of-bias results were visualized using the online Robvis tool (https://mcguinlu.shinyapps.io/robvis/) (McGuinness and Higgins, 2020). As no visualization tool exists for ROBINS-I V2, the ROBINS-I template was adopted due to its consistent rating scale.

### 2.5 Statistical Analyses

This review combined quantitative and qualitative approaches to achieve a comprehensive and systematic analysis of the included studies.

For quantitative synthesis, statistical analyses were conducted using Comprehensive Meta-Analysis software (CMA, version 3.3). First, Egger’s test was applied within CMA to assess publication bias in subgroups that met the minimum number of studies. Reported statistics (*F*, *t*, means, and standard deviations) were then converted into correlation coefficients using Fisher’s Z transformation (Zheng and Peng, 2001). Effect sizes were interpreted by transforming the results back into correlation coefficients (*r*). Only one effect size per unique sample, per brain region was used in the meta-analysis to ensure independence, and ROI (region of interest)-based extraction was applied to distinguish different brain regions. Due to the lack of standardized spatial coordinates (e.g., MNI coordinates) in many studies, we adopted a systematic ROI-based aggregation approach. Anatomical targets were defined as broad ROIs, allowing the inclusion of studies with varying levels of spatial precision and facilitating quantitative synthesis across a wider evidence base. Following current best practices in meta-analysis, all subgroups were analyzed under a random-effects model regardless of heterogeneity test results, as heterogeneity was assumed to exist between studies (Borenstein et al., 2021). In this framework, CMA automatically assigns weights to each study based on the inverse of its variance, ensuring that studies with more precise estimates contribute more to the overall effect. Heterogeneity statistics ($I^{2}$, ${\mathrm{Tau}}^{2}$, Cochran’s Q tests) were calculated and reported for descriptive purposes only.

For the qualitative synthesis, a thematic analysis with an inductive and semantic orientation was employed to analyze findings from 17 studies——effects of SBIs on children’s neural functioning. Following the six-phase procedure (Braun and Clarke, 2006), we initiated the process by extracting outcome-related textual content from the studies. Building upon this foundation, meaningful lexical units were identified and inductively coded, which gradually coalesced into higher-order conceptual categories. Throughout this analytical journey, emergent themes were continuously refined through critical reappraisal to safeguard their conceptual distinctiveness. The analysis ultimately yielded a set of overarching themes that represent the principal mechanisms through which SBIs, designed with different instructional purposes, exert effects on children’s neural functioning. These themes provide the structure of the results section, where each is presented with supporting evidence and interpretation.

## 3 Results

### 3.1 Studies characteristic

A total of 17 studies met the inclusion criteria for this review. These studies collectively involved 912 participants, with an average sample size of 54 (range: 12–93). Eight studies originated from China (47.1%), two from the United States (11.8%), and one each from France, Israel, Norway, Brazil, and Spain (5.9% each), with two from the Netherlands (11.8%).

Five studies (29.4%) focused on children aged six years or younger, while twelve (70.6%) examined children aged 6–12 years. Regarding neuroimaging modalities, seven studies used functional near-infrared spectroscopy (fNIRS; 41.2%), four used electroencephalography (EEG; 23.5%), five employed magnetic resonance imaging (MRI; 29.4%), and one combined magnetoencephalography (MEG) with functional MRI (fMRI; 5.9%). By publication year, seven studies were published in 2024 (41.2%), two in 2023 (11.8%), one in 2022 (5.9%), three in 2021 (17.6%), three in 2019 (17.6%), and one in 2014 (5.9%).

Based on their primary learning objectives, studies were classified into five categories: executive function enhancement (EF; *k* = 9), stress regulation improvement (SR; *k* = 2), language skills development (LS; *k* = 3), auditory function development (AF; *k* = 1), and investigations focusing on the SBIs process itself (IP, *k* = 2). Appendix A, B and C summarize the study characteristics including basic information and research methods, while Fig. 2 illustrates the brain regions reported across the included studies.

**Fig. 2:**
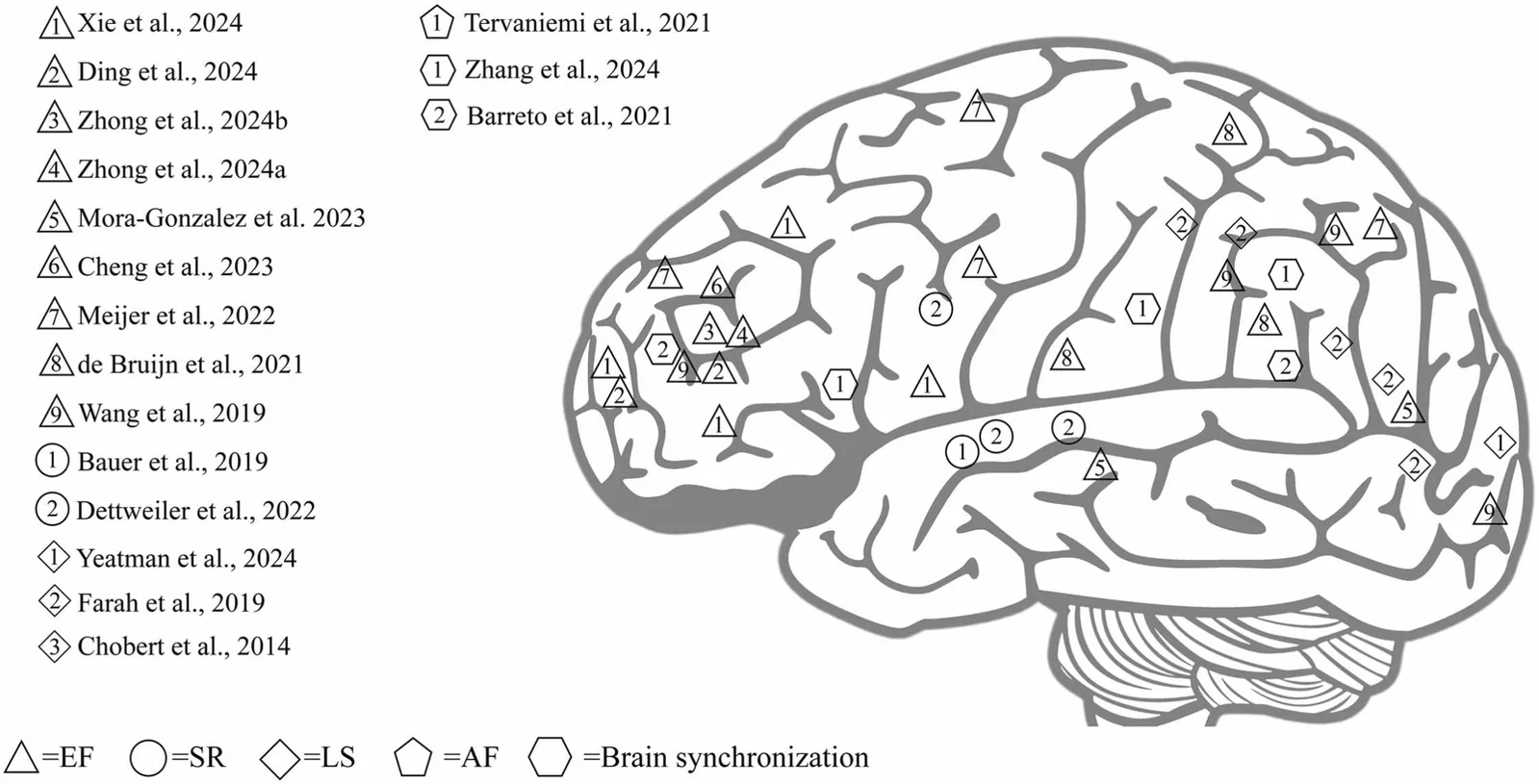
*The effect of learning on the function of brain regions. Note*. Two EEG studies didn’t mention the specific brain regions affected, therefore they were not presented in the figure (Chobert et al., 2014, Tervaniemi et al., 2021).

### 3.2 Cochrane Risk of Bias

The risk of bias was assessed using the RoB 2 tool for the 15 randomized controlled trials and the ROBINS-I V2 tool for the 2 non-randomized studies (Fig. 3, Fig. 4). Most studies demonstrated a low risk of bias in intervention implementation and data analysis. However, some randomized trials lacked sufficient detail on randomization procedures, and one study from each design was rated as high risk due to inadequate methodological description. Although these high-risk studies present certain limitations, they still offer meaningful insights and were therefore retained for subsequent analyses to ensure a comprehensive synthesis of the available evidence.

**Fig. 3:**
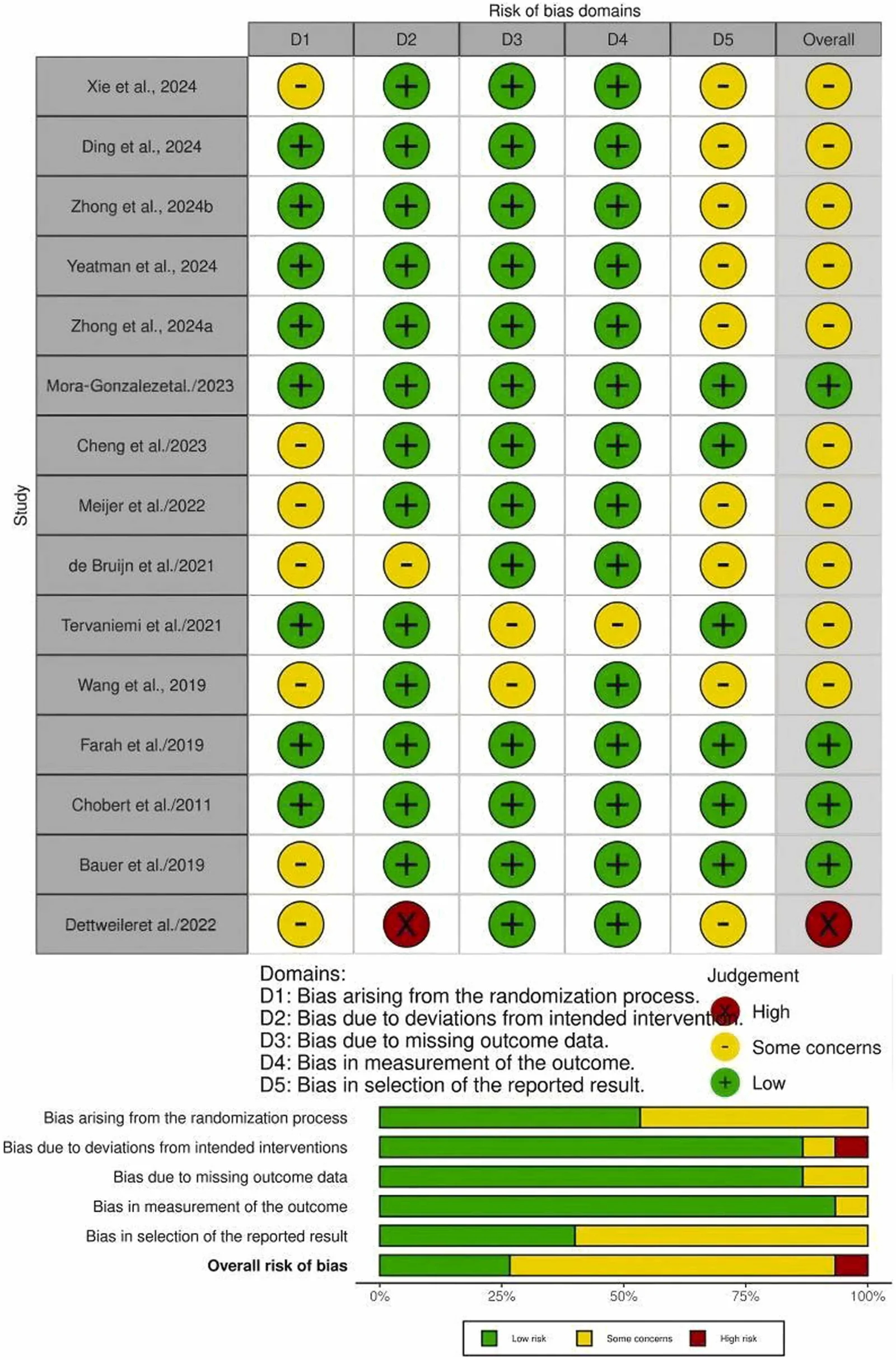
Risk of bias for 15 RCT studies.

**Fig. 4:**
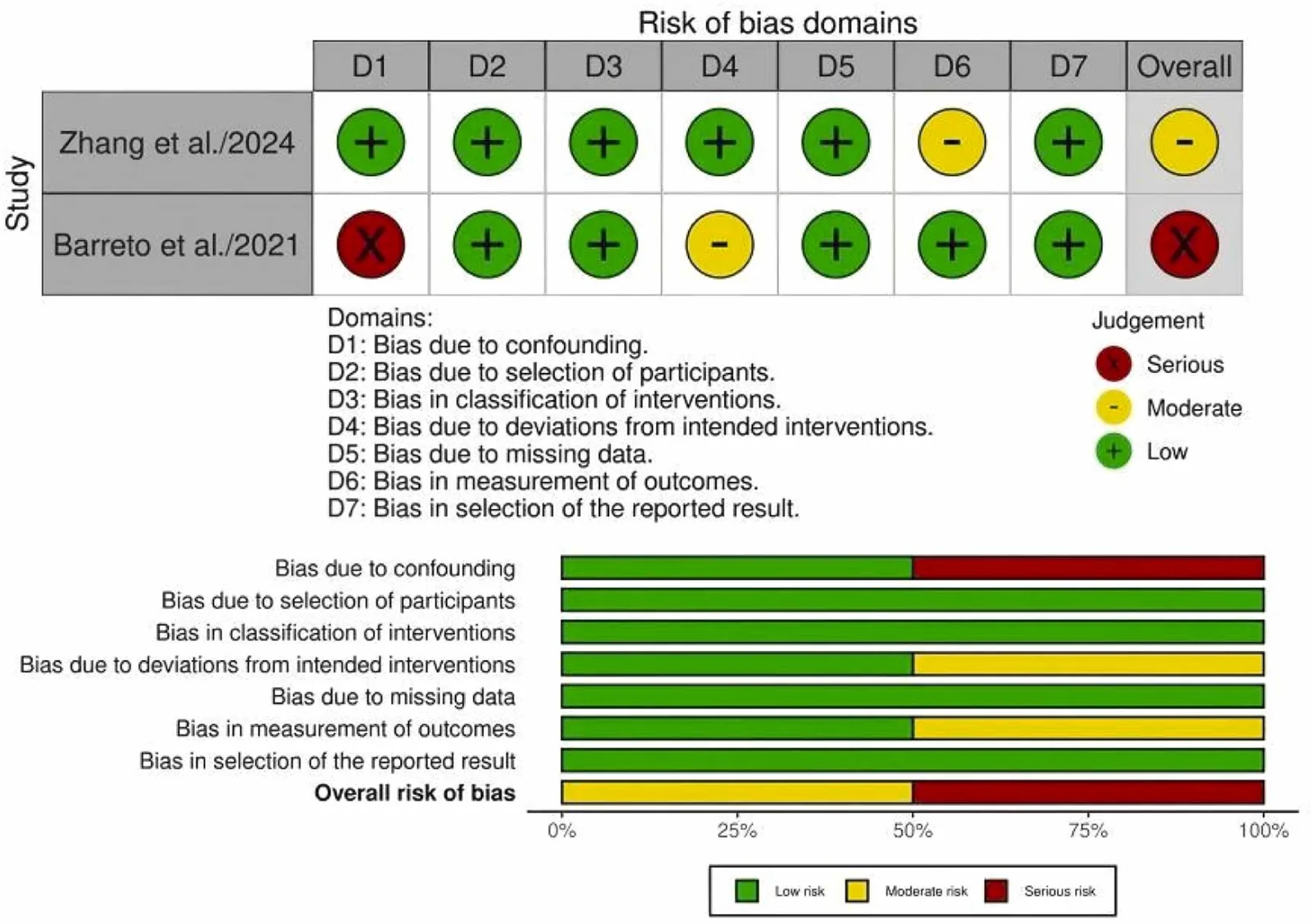
Risk of bias for 2 non-randomized studies.

### 3.3 Association of SBIs with Neural Development

To address RQ1, a meta-analysis was conducted to examine the association between different SBIs and neural development outcomes in children aged 3–12. The included studies were first categorized by their primary learning objectives into five groups: executive function enhancement (EF), stress regulation improvement (SR), language skill (LS), auditory function development (AF), and SBIs process (IP).

Several studies and subgroups were excluded from the quantitative synthesis. First, two studies focusing on LS were excluded due to a lack of overlapping brain regions across studies, which is a prerequisite for coordinate-based meta-analysis. Furthermore, although the AF and IP subgroups were identified, each contained only a single study, precluding a meaningful meta-analysis; thus, these subgroups were also excluded. Additionally, five studies were excluded owing to inconsistent neuroimaging methodologies and incomparable data reporting.

For the remaining studies, as most did not provide MNI coordinates, a region-of-interest (ROI) approach was employed. Specifically, effect sizes for EF-related studies were extracted from the prefrontal cortex (PFC), and for SR-related studies from the amygdala.

Therefore, the remaining 8 studies (N = 432 participants) were included in the quantitative synthesis. For each category, we conducted publication bias analysis, heterogeneity testing, and effect size estimation.

Only the EF subgroup met the minimum number of studies required for Egger’s regression test for publication bias (*k* ≥ 3). Therefore, Egger’s test was conducted for the EF subgroups. For other subgroups, the small number of studies precluded meaningful interpretation of funnel plot asymmetry or Egger’s regression, and thus publication bias was not assessed. For EF, the funnel plot showed effect sizes symmetrically distributed around the pooled effect, with non-significant intercepts in the results (Fig. 5). Specifically, the intercept for EF was - 6.627 (*Egger’s* t = 0.515, *SE* = 12.851, *p* = 0.633). In general, there was no evidence that the current meta-analysis was substantially affected by publication bias.

**Fig. 5:**
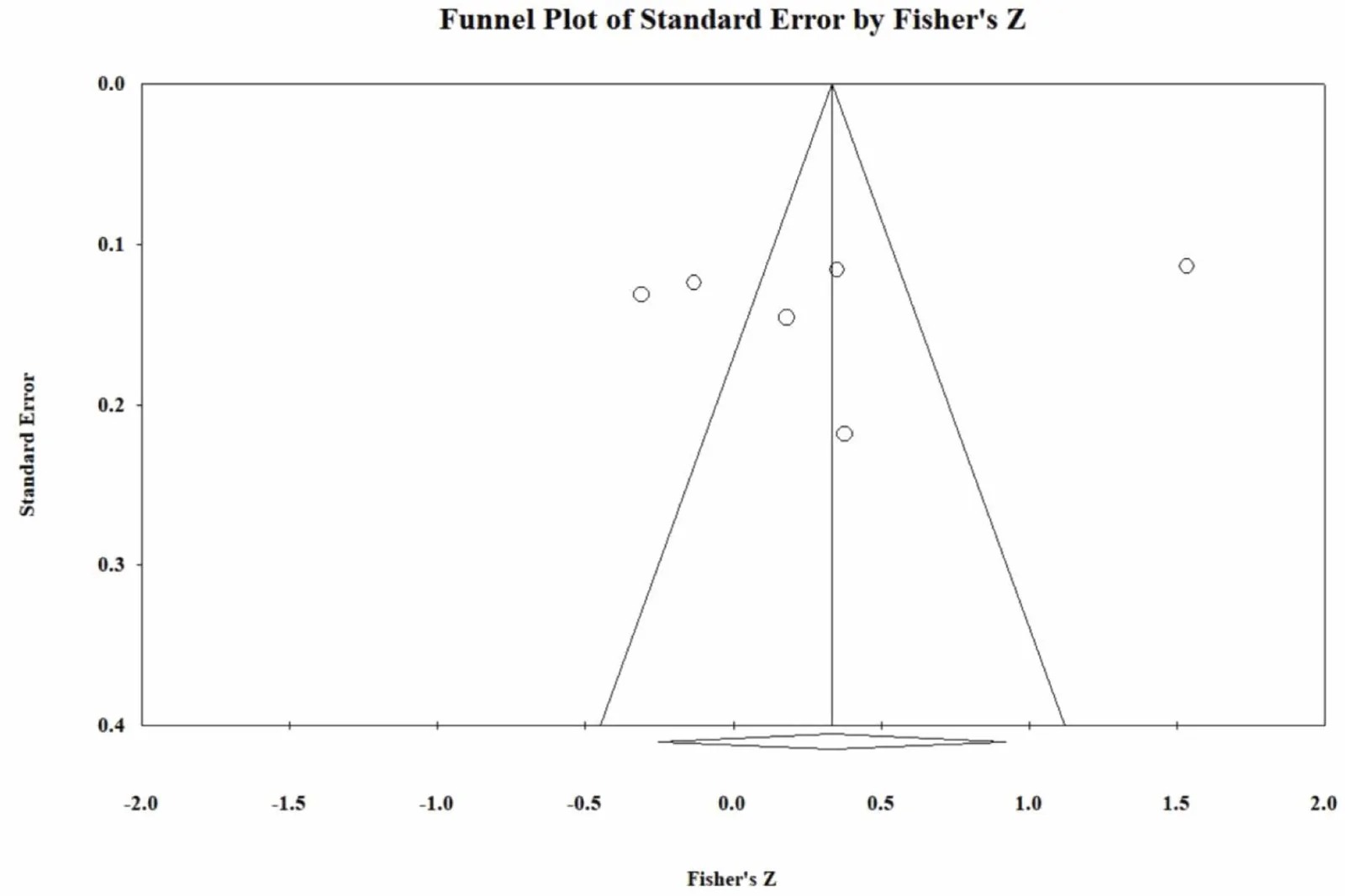
Funnel plot of the association between the development of EF and SBIs.

Heterogeneity was assessed using Cochran’s Q and the *I²* statistic (Table 2). As shown in Table 5, only EF (*p* < 0.01) exhibited statistically significant heterogeneity, indicating substantial variability in effect sizes across studies and supporting the use of a random-effects model to estimate the overall effect. In contrast, SR (p > 0.05) did not show significant heterogeneity. However, existing research suggests that heterogeneity is typically expected in psychological and neuroimaging data and cautions against selecting between fixed- and random-effects models based solely on statistical tests (I²/Q) (Borenstein et al., 2021). Therefore, a random-effects model was applied for both EF and SR analyses.

**Table 2:** Heterogeneity test results for each category.

Category	*k*	*n*	Q-test		$I^{2}$	Tau-squared	
			*Q*	*df(Q)*		*Tau*	${\mathrm{Tau}}^{2}$
EF	6	360	148.991**	5	96.643%	0.719	0.516
SR	2	72	1.099	1	9.007%	0.058	0.003

*Note.* **p＜0.01; *k* = number of studies, *n* = number of participants; *Tau*=standard deviation of true effects; Random-effects model applied to all subgroups by default; heterogeneity statistics are descriptive only.

Pooled effect sizes for each category are summarized in Table 3. SBIs were negatively associated with neural outcomes related to stress regulation (SR) (*r* = -0.277, 95% CI [-0.502, −0.017], *p* < .05), but no significant associations were observed for executive function (EF) (*r* = 0.322, 95% CI [-0.247, 0.726], *p* > .05), as the confidence interval encompassed zero. The pooled effect size for EF was non-significant, with substantial heterogeneity observed in both the magnitude and direction of effects. This variability likely represents a complex distribution of neural responses across the included studies. To further explore these divergent patterns, a thematic analysis was conducted, allowing for a qualitative characterization of mechanisms such as neural recruitment and efficiency. Given the limited study count (*k* = 6), further quantitative sub-grouping was not undertaken because it would have produced underpowered and statistically unstable estimates.

**Table 3:** Overall effect size of associations between each category and students’ brain function.

Category	*k*	*n*	*r*	95% CI		*Z*
				lower	upper	
EF	6	360	0.322	-0.247	0.726	1.117
SR	2	65	-0.277*	-0.502	-0.017	-2.085

*Note.* *p＜0.05; *k* = number of studies, *n* = number of participants. AF and IP categories were excluded from the meta-analysis as they each contained only a single study, precluding pooled estimation.

The pooled SR effect size fell within the moderate range according to the benchmarks proposed by Lipsey and Wilson (2001). However, this finding should be interpreted as a preliminary trend because only two studies were included in this subgroup (*k* = 2), and one of which was rated as having a high risk of bias. Due to the limited number of studies, sensitivity analysis was not feasible. Although both studies showed consistent effect directions, additional research is required before any reliable conclusion can be drawn. Forest plots for SR are presented in Fig. 6.

**Fig. 6:**
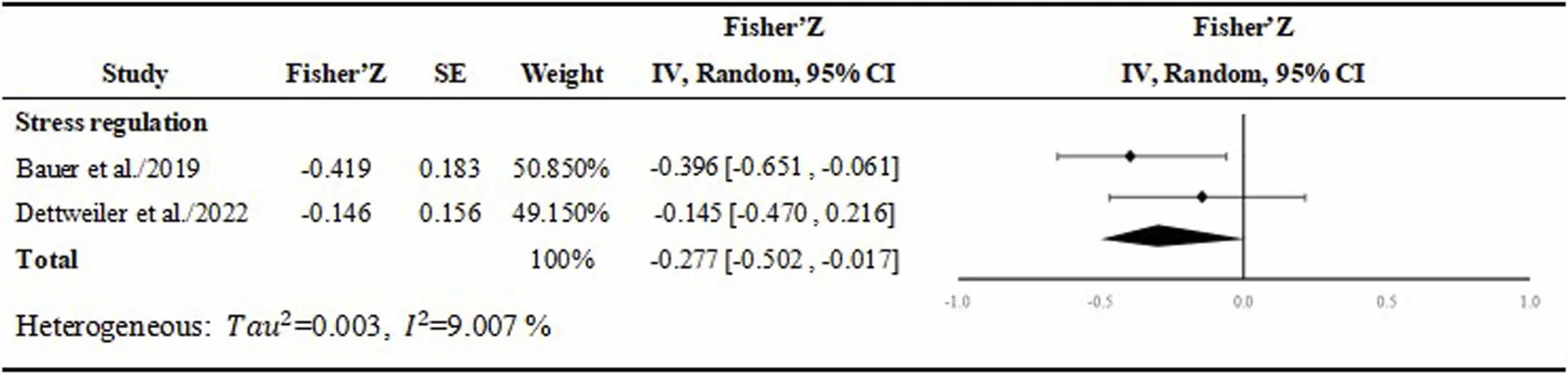
Forest plot of the correlation between SR and student brain function development.

### 3.4 Neural mechanisms underlying SBI effects

A thematic analysis of all 17 included studies was conducted to address the second research question regarding the neural mechanisms of SBIs. This qualitative synthesis revealed four distinct but complementary themes, illustrating how interventions targeting similar developmental goals can operate through diverse neural pathways (Table 4). To ensure the robustness of these findings, we performed a qualitative sensitivity check by comparing neural patterns across study quality levels (Dixon-Woods et al., 2006, Thomas and Harden, 2008). This comparison indicated that the main themes identified in the synthesis were also supported by low-risk studies, suggesting that the overall interpretation was not solely dependent on studies rated as high risk of bias.

**Table 4:** Thematic Coding of SBI Neural Mechanisms.

**Theme**	**Effect Direction**	**Brain Hemisphere**		**Intervention Content**		**Behavioral Outcomes**		**SBI-Related Neural Changes**	**Authors / year**
Recruitment of Domain-Specific Executive Networks	↓	Bilateral		mindfulness-based intervention		Executive function		Activation of BA 6, 8, 9, 10, 46	Xie et al., (2024)
				engineering STEM learning		Executive function		Activation of BA 10 and 46	Ding et al., (2024)
				Exercise intervention		Inhibitory control		Prefrontal Current Density	Mora-Gonzalez et al., (2023)
	↑	Left		Volleyball training		Working memory		Activation of L-DLPFC	Zhong et al., (2024b)
		Bilateral		Exercise intervention		Working memory		Prefrontal Current Density	Mora-Gonzalez et al., (2023)
		Right		Physical training		Inhibitory control		Activation of R-DLPFC	Zhong et al., (2024a)
				Equine-Assisted Activities		Inhibitory control			Cheng et al., (2023)
				Abacus-Based Mental Calculation		Visuospatial working memory		Frontal, parietal, and occipital regions (BA7, 17, 18, 40)	Wang et al., (2019)
	↔	/		Aerobic exercise intervention		/		/	Meijer et al., (2022)
				Aerobic versus cognitively-engaging physical activity		Visuospatial working memory			de Bruijn et al., (2021)
Modulation of Emotion Regulation Circuits	↑	Bilateral		Mindfulness training vs code training		Stress perception and regulation		Connections between the right amygdala and the ventromedial PFC	Bauer et al., (2019)
				outdoor education project		Biological stress-reactivity		Maturation of ACC	Dettweiler et al., (2022)
	↓	Right		Mindfulness training vs code training		Stress perception and regulation		Activation of amygdala	Bauer et al., (2019)
	↔	/		outdoor education project		Biological stress-reactivity		Hippocampus	Dettweiler et al., (2022)
Sensory-Specialized Adaptation in Language and Auditory Processing	↑	Bilateral		Letter intervention VS Language intervention		/		Activation and structural changes of VMFA	Yeatman et al., (2024)
				Music Versus Foreign Language Training		/		Time and group effects of MMN in music group Lager P3a in English group	Tervaniemi et al., (2021)
				Active music training		/		Lager amplitudes of MMN in music group	Chobert et al., (2014)
				screens vs dialogic reading (DRG)		Narrative comprehension		Network efficiency in DRG	Farah et al., (2019)
Preliminary Evidence between Teacher-Student Inter-Brain Synchrony	↑	Bilateral	traditional teaching, storyboard teaching, story-based teaching		Learning performance		The level of brain synchronization in the upper IFG		Zhang et al., (2024)
			Analysis method		/		Functional connectivity of PFC and TPJ		Barreto et al., (2021)

*Note*. DLPFC=dorsolateral prefrontal cortex; TPJ=temporoparietal junction; MMN=mismatch negative; IFG=inferior temporal gyrus; ACC=anterior cingulated cortex; VMFA=visual word form area; BA=Brodmann area; R-=right-, L-=left-; ↔=none effect;↑=stronger activation;↓=lower activation.

Themes 1 through 3 characterize pathways of long-term neural plasticity within the individual. In contrast, Theme 4 identifies a distinct neural mechanism underlying the quality of pedagogical interaction and proximal learning engagement, serving as a dyadic marker of the effective process of intervention delivery.

#### 3.4.1 Theme 1: a dual-pathway model of executive network recruitment

Nine studies examining EF-focused SBIs consistently implicated a network of brain regions including the dorsolateral prefrontal cortex (dlPFC; BA9, 46), frontopolar cortex (BA10), and parietal cortices. The findings suggest a dual-pathway model, where the neural response depends on the nature of the intervention.

The first pathway, characterized by ‘increased neural recruitment’, was observed in studies of cognitively demanding physical activities. For example, interventions such as volleyball training (Zhong et al., 2024b), physical fitness training (Zhong et al., 2024a), and equine-assisted activities (Cheng et al., 2023) led to greater activation in the dlPFC. This pattern suggests that these activities engage executive control networks more robustly to meet complex task demands.

The second pathway, reflecting ‘enhanced neural efficiency’, was characterized by ‘decreased’ prefrontal activation following sustained practice. This was reported for knowledge-based interventions, including mindfulness training (Xie et al., 2024) and a design-based STEM curriculum (Ding et al., 2024). This reduction in activation is interpreted as the brain performing the same cognitive task more efficiently and with fewer neural resources after training. Notably, two studies of aerobic exercise without a clear cognitive-scaffolding component reported null effects (de Bruijn et al., 2021, Meijer et al., 2022), underscoring that changes in executive networks are tied to specific types of engagement.

#### 3.4.2 Theme 2: Convergent Modulation of Emotion Regulation Circuits

Two studies investigating stress regulation interventions both identified changes within prefrontal-limbic circuits, but revealed different mechanisms converging on a similar outcome. One study on mindfulness training reported functional changes: decreased right amygdala reactivity to threat cues, coupled with strengthened functional connectivity between the amygdala and the ventromedial prefrontal cortex (vmPFC) (Bauer et al., 2019). This pattern is indicative of enhanced top-down regulation of emotional responses. In contrast, observations from an outdoor education program suggested a potential link to the structural maturation of the anterior cingulate cortex (ACC), which was associated with reduced biological stress reactivity (Dettweiler et al., 2022). Together, these studies tentatively suggest that SBIs targeting stress regulation may involve multiple neural processes, including functional prefrontal regulation of the amygdala and, potentially, structural development in regulatory regions such as the ACC. Given the small number of studies and variation in study quality, these interpretations should be considered preliminary.

#### 3.4.3 Theme 3: Hierarchical Adaptation in Language and Auditory Processing

Four studies examining language and auditory interventions revealed a hierarchical pattern of neural adaptation, affecting both low-level sensory processing and higher-order cognitive integration. At the pre-attentive level, music training consistently enhanced the amplitude of the mismatch negativity (MMN) response to auditory deviants, indicating more precise neural encoding of features like pitch and duration in the auditory cortex (Chobert et al., 2014, Tervaniemi et al., 2021).

At a higher cognitive level, interventions engaged attentional and specialized processing networks. Foreign language training, for example, elicited a larger P3a component than music training, suggesting greater recruitment of attentional resources for stimulus evaluation (Tervaniemi et al., 2021). In the domain of reading, dialogic reading was associated with greater whole-brain network efficiency (Farah et al., 2019), while systematic phonics instruction was specifically linked to increased activation and specialization of the visual word form area (VWFA) (Yeatman et al., 2024). These findings suggest that language-focused SBIs induce plasticity at multiple stages of the processing hierarchy, from refining early auditory discrimination to optimizing higher-order cortical networks for attention and reading.

#### 3.4.4 Theme 4: Inter-Brain Synchrony as a Marker of Pedagogical Interaction

Two hyperscanning studies provided preliminary evidence for a dyadic neural mechanism in classroom learning. These studies reported significant inter-brain synchrony or neural coupling between teachers and students in regions associated with social cognition and language, including the inferior frontal gyrus (IFG) and temporoparietal junction (TPJ) (Barreto et al., 2021, Zhang et al., 2024). Notably, IBS in the IFG was found to be positively correlated with students’ STEM learning performance (Zhang et al., 2024), suggesting that this neural alignment transcends simple shared sensory input and relates to functional pedagogical outcomes. Although based on a small number of studies, these findings suggest that effective teaching and learning may be supported by the dynamic alignment of neural activity between teacher and child.

## 4 Discussion

This systematic review and exploratory meta-analysis integrated neuroimaging evidence to elucidate how SBIs influence children’s neurodevelopment. The findings reveal a critical distinction: while a quantitative synthesis points to domain-specific effects, a qualitative analysis is necessary to understand the diverse and often opposing neural mechanisms that underlie them. Specifically, our meta-analysis identified a preliminary pooled estimate suggestive of reduced amygdala activity following SBIs; however, given the inclusion of only two studies, this result should be treated as exploratory rather than confirmatory. The non-significant pooled effect for executive function is therefore interpreted primarily as a reflection of extreme heterogeneity across studies, rather than as evidence of a null effect. Our thematic synthesis resolves this ambiguity by demonstrating that different interventions can achieve similar behavioral goals through multiple, distinct neural pathways. These findings collectively advance a more nuanced understanding of educational plasticity, moving beyond whether interventions work to how they shape the developing brain.

### 4.1 Domain-specific and heterogeneous neural effects of SBIs

Our first research question addressed the overall strength of association between SBIs and neural outcomes. The meta-analysis revealed a clear, domain-specific pattern. Interventions targeting stress regulation (SR) showed a potential association with changes in brain function, characterized by an observed trend of reduced amygdala activity during the stress regulation tasks. This was quantitatively reflected in our initial meta-analytic estimate (*r* = –.277), suggesting a link between the intervention and an attenuated amygdala response during stress regulation. While the small number of studies (*k* = 2) necessitates caution, this finding aligns with established theoretical models. The amygdala is a central hub for threat detection and emotional arousal (LeDoux, 2013), and its attenuated activity is a widely accepted marker of improved emotional control (Desbordes et al., 2012). The emergence of this effect in the current sample, albeit preliminary, suggests that interventions like mindfulness and nature exposure may engage this core limbic circuit. Critically, these preliminary results point toward the possibility that the prefrontal-amygdala circuitry, despite its protracted maturation (Gee et al., 2013), possesses significant functional plasticity in childhood and is responsive to targeted environmental input.

In contrast, the meta-analysis for executive function (EF) yielded a non-significant overall effect, a result driven by exceptionally high heterogeneity (*I²* = 96.6%). This null finding should not be interpreted as evidence of ineffectiveness. Rather, it is a statistical artifact of a profound underlying principle: EF interventions operate through at least two opposing neural mechanisms. Although isolating these mechanisms through quantitative sub-grouping would be theoretically ideal, the limited sample size (*k* = 6) would have rendered such statistical partitioning underpowered and statistically unstable (Higgins et al., 2019). Consequently, this extreme heterogeneity is treated here as a primary discovery that reflects the functional complexity of EF rather than a statistical failure (Huedo-Medina et al., 2006).

Following the framework of Thomas and Harden (2008), we used thematic synthesis to characterize these divergent patterns more appropriately than a single pooled model could. As our thematic analysis revealed, some interventions, particularly those involving cognitively demanding physical activity, lead to increased prefrontal activation (neural recruitment), while others, such as those based on sustained practice like STEM or mindfulness, lead to decreased activation (neural efficiency). When pooled in a meta-analysis, these opposing effects cancel each other out, producing a null result (Borenstein et al., 2021). This insight is critical, as it explains the well-documented gap between consistent behavioral improvements in EF and inconsistent neural findings. The quantitative results, therefore, answer the “what” of our first research question while powerfully motivating the “how” of the second.

### 4.2 Multiple neural pathways to common developmental goals

Our second research question sought to identify the specific neural mechanisms underlying SBI effects. The thematic analysis revealed a core principle of educational neuroscience: multiple realizability, where different interventions can produce similar developmental outcomes through distinct neural pathways. Notably, although the small number of studies precluded a quantitative sensitivity analysis within the meta-analytic model, we conducted a qualitative comparison of findings across study quality levels (Dixon-Woods et al., 2006, Thomas and Harden, 2008). This comparison suggested that the core neural patterns discussed in the synthesis were also present in low-risk studies, ensuring that the synthesis was not disproportionately driven by methodologically weaker evidence.

The dual-pathway model of EF development is the clearest example. Physically-oriented SBIs that introduce novel rule-sets appear to work by enhancing neural recruitment, strengthening the engagement of executive control networks to manage cognitive load (Diamond, 2013). In contrast, practice-based SBIs appear to work by promoting neural efficiency, allowing the brain to perform tasks with fewer cognitive resources (Brooks et al., 2020). Thus, educators can foster EF through activities that either build the capacity to recruit cognitive control or refine the efficiency of its deployment.

This principle extends to other domains. Interventions for stress regulation, for example, converge on rebalancing the limbic-prefrontal circuit through different means. Mindfulness training appears to enhance top-down functional connectivity between the prefrontal cortex and the amygdala (Bauer et al., 2019), while immersive outdoor education may promote the structural maturation of regulatory hubs like the anterior cingulate cortex (Dettweiler et al., 2022). Similarly, language and auditory interventions exhibit a hierarchical mechanism, enhancing both low-level pre-attentive sensory processing (evidenced by MMN changes) and higher-order cognitive integration and specialization (evidenced by P3a and VWFA changes).

Finally, the nascent evidence on teacher-student inter-brain synchrony introduces another level of analysis. It suggests that the mechanisms of learning are not confined to the individual brain but are also dyadic and interactive. The finding that neural coupling in the inferior frontal gyrus correlates with learning outcomes points to a neurobiological marker of effective pedagogy, reflecting shared attention and cognitive alignment (Dikker et al., 2017). These findings underscore that a comprehensive model of educational plasticity must account for mechanisms operating at the functional, structural, and even interpersonal levels.

### 4.3 Limitations and future directions

This review, while offering a novel synthesis, is subject to limitations inherent to a young and developing field. First, the small number of included studies (N = 17) and the even smaller number in each meta-analytic subgroup limit the statistical power and generalizability of our quantitative findings. In particular, the stress regulation (SR) subgroup, consisting of only two studies, yielded results that are methodologically precarious and highly susceptible to bias. These findings should be treated as preliminary trends rather than definitive evidence. The field urgently needs larger, multi-site, and more diverse studies to build a robust evidence base.

Second, methodological heterogeneity, particularly the lack of standardized coordinate reporting (e.g., MNI space), precluded more precise coordinate-based meta-analyses and forced a broader region-of-interest approach. By pooling effect sizes across functionally distinct subregions within each ROI, the analysis necessarily combines signals from anatomically and functionally heterogeneous areas. As a result, the quantitative findings reflect broad regional patterns rather than subregion-specific effects, limiting the anatomical specificity of the conclusions. This limitation is inherent to the available data, rather than a modeling choice. Importantly, this structural limitation is distinct from the functional heterogeneity addressed in the thematic analysis, which captures variability in cognitive processes and intervention types rather than spatial resolution. Future research should adhere to standardized reporting guidelines to facilitate more granular and reliable cross-study integration.

The predominance of fNIRS studies (41.2%) may introduce a spatial bias toward cortical mechanisms, particularly within the prefrontal cortex. As fNIRS is primarily sensitive to cortical activity and has limited ability in detecting subcortical or deeper structural changes, the current findings may overemphasize cortical hemodynamic responses while under-representing deeper neural processes, and should therefore be interpreted with caution.

Finally, most studies focused on single neural indicators within specific domains. This may underestimate the full impact of SBIs, which often engage multiple interacting neural systems (e.g., cognitive-emotional interactions). Future work should leverage multimodal neuroimaging and complex task designs to capture these network-level dynamics more effectively.

### 4.4 Implications for theory and practice

Despite these limitations, this review offers important implications. Theoretically, our findings champion a shift from asking whether interventions work to understanding how they work. By highlighting the principle of multiple neural realizability, this review enriches our models of brain plasticity, demonstrating that there is no single “correct” way to influence a developing neural system.

Practically, this understanding can inform more purposeful educational design. First, educators can select or combine interventions based on their intended neural mechanism—for instance, choosing activities that promote neural efficiency for skill consolidation versus those that enhance neural recruitment for tackling novel problems. Second, the evidence on inter-brain synchrony provides a neurobiological basis for what effective teachers have long known: high-quality, socially attuned interaction is fundamental to learning. Finally, neuroimaging tools, while not yet ready for individual classroom diagnostics, offer objective, “in-vivo” methods for evaluating intervention effectiveness and the quality of pedagogical interactions, connecting educational practice directly to the brain’s developmental processes.

## 5 Conclusion

This systematic review and exploratory meta-analysis provides a cohesive synthesis of how school-based interventions shape children’s neurodevelopment. The evidence reveals that SBIs produce domain-specific neural effects, but their mechanisms are diverse and multifaceted. Interventions for stress regulation consistently modulate amygdala activity, while those for executive function operate via a dual pathway of neural recruitment and efficiency. Language and auditory programs engage a hierarchical system of sensory and cognitive processing, and emerging evidence suggests that learning is further supported by teacher-student neural synchrony. A comprehensive understanding of educational neuroscience requires integrating these multiple levels of analysis—from cellular plasticity to interpersonal neural dynamics. This framework not only consolidates current knowledge but also lays the foundation for designing more precise and effective educational experiences to support the holistic development of every child.

## CRediT authorship contribution statement

**Runke Huang:** Writing – review & editing, Writing – original draft, Visualization, Validation, Methodology, Formal analysis, Data curation. **Jingwen Li:** Writing – review & editing, Writing – original draft, Supervision, Resources, Project administration, Investigation, Funding acquisition, Conceptualization. **Hui Li:** Writing – review & editing, Writing – original draft, Supervision, Project administration.

## Declaration of Generative AI and AI-assisted technologies in the writing process

During the preparation of this work, the authors used AI-empowered Grammarly in order to clean grammatical errors and to improve its readability. After using this tool, the authors reviewed and edited the content as needed and take full responsibility for the content of the publication.

## Declaration of Competing Interest

The authors declare that they have no known competing financial interests or personal relationships that could have appeared to influence the work reported in this paper.

## Figures and Tables

**Table:** 

**Authors/year**	**Country**	**Participants**		**Lab/ Natural**	**Active/Passive Learning**
		**Sample size and age**	**Inclusion criteria**		
Xie et al., (2024)	China	N = 68 (male=41) Age range= 4–6 yr. Mage= 61.8 ± 10.7 months	/	lab + nature course	Passive
Ding et al., (2024)	China	N = 61 (male=45) Mage= 10.74 ± 0.44 yr.	low SES families; staying at the school during weekdays; have normal development; no medical or psychiatric history	lab + nature course	Active
Zhong et al., (2024b)	China	N = 80 (male=44) Mage= 9.59 ± 1.18 yr.	physically healthy; normal or corrected normal vision without color blindness; right-hander	lab + nature course	Passive
Yeatman et al., (2024)	America	N = 48 (male=19) Age range= 4y10m to 6y2m Letter Group: N = 24 Language Group: N = 24	No any developmental disorder; normal or corrected-to-normal vision	lab + nature course	Passive
Zhong et al., (2024a)	China	N = 80 (male=39) EG: N = 40 (male=19) CG: N = 40 (male=20)	Physical health; Normal or corrected vision, no color blindness, color weakness or other eye diseases; Right-hander; Normal intelligence.	lab + nature course	Passive
Mora-Gonzalez et al., (2023)	Spain	N = 67 (male=41) Mage= 10.00 ± 1.10 yr.	having completed the preand post-intervention EEG and both task assessments; having attended at least 70% of the required three sessions/week; obesity	lab + nature course	Passive
Cheng et al., (2023)	China	N = 48, Age range= 7–8 yr. IG: N = 24 (male=12) Age range= 7–8 yr. CG: N = 24 (male=12) Age range= 7–8 yr.	(1) Inclusion: normal intelligence, no cognitive disorders, right-hander, no horse-riding training in the last 6 months (2) Exclusion: BMI ≥ 24, motor or physical disability, in multiple sports training on weekends, no history of horsehair allergies, fear of horses	lab + nature course	Passive
Meijer et al., (2022)	Netherlands	N = 93, Age range= 9–10 yr.	no contraindications for MRI	lab + nature course	Passive
de Bruijn et al., (2021)	Netherlands	N = 62 (male=30) Mage= 9.2 ± 0.61 yr.	/	lab + nature course	Passive
Tervaniemi et al., (2021)	China	English: N = 38, Mage= 9.18 ± 0.75 yr. Music: N = 29, Mage= 9.42 ± 0.8 yr.	/	lab + nature course	Passive
Wang et al., (2019)	China	Abacus-based mental calculation: N = 27 (male=14) CG: N = 23 (male=12) Age range= 10–12 yr.	Urban family, normal hearing, normal vision or correction No reports of neurological or psychiatric disorders There are no special educational assistance requirements either	lab + nature course	Passive
Farah et al., (2019)	Israel	DRG: N = 16, Mage= 64.35 ± 3.99 months SSG: N = 16, Mage= 58 ± 6 months	From a medium class background, right-hander Normal or corrected binocular vision, Normal hearing Normal neurological and developmental history	lab + nature course	Passive
Chobert et al., (2014)	France	Music: N = 12 (male=9) Mage= 8.3 ± 0.45 yr. Painting: N = 12 (male=4) Mage= 8.2 ± 0.45 yr.	none of the children, and none of their parents, had formal training in music or painting.	lab + nature course	Passive
Bauer et al., (2019)	America	IG: N = 18 (male=3) Mage= 12.12 ± 0.43 yr. CG: N = 15(male=6) Mage= 11.92 ± 0.36 yr.	Exclude children who engage in excessive exercise and wear braces	lab + nature course	Passive
Dettweiler et al., (2022)	Norway	IG:N = 37 CG:N = 11 Age: 5th and 6th grade	/	lab + nature course	Passive
Zhang et al./(2024)	China	N = 39 Mage= 6.90 ± 0.48 yr.	/	natural	Active
Barreto et al./(2021)	Brazil	Adults: N = 4 (male=2) Age range= 21–28 yr. Children: N = 8 (male=4) Age range= 3–5 yr.	/	natural	Active

*Note.* IG=Intervention group; CG=Control group; DRG=dialogic reading group; SSG=screen story group; AMC=Abacus-based mental calculatio; UNI= Unidirectional; DIA=Dialogue; COL=Collaborative; STU=Student-led.

**Table:** 

**Authors/year**	**Method**					
	**Neuroimaging**	**Brain region**	**Intervention content**	**Experimental type**	**Ability to intervene**	**Intervention duration**
Xie et al., (2024)	fNIRS	PFC	Mindfulness-based Class	RCT	executive function	20 min per session, twice a week, all together 15 sessions
Ding et al., (2024)	fNIRS	PFC	Reverse engineering STEM learning	RCT	executive function	2 weeks, twice a week, 1 h each time
Zhong et al., (2024b)	fNIRS	PFC	Volleyball training	RCT	working memory	2 weeks, twice a week, 1 h each time
Yeatman et al., (2024)	MEG, fMRI	visual cortex	Letter intervention Language intervention	RCT	reading skill	2 h per week, 5 years
Zhong et al., (2024a)	fNIRS	PFC	Physical training	RCT	inhibitory control	60 min per session, 3 times a week, 12 weeks
Mora-Gonzalez et al., (2023)	EEG	whole brain	IG: Aerobic exercise. CG: Maintain regular exercise.	RCT	executive function	90 min/session, 5 sessions/week, 2 weeks
Cheng et al., (2023)	fNIRS	PFC motor cortex	IG: equine-assisted activity CG: daily growth	RCT	inhibitory control	45–55 min per session, twice a week, 12 weeks
Meijer et al., (2022)	fMRI	whole brain	Aerobic exercise Cognitively demanding exercise Control group: routine exercise	RCT	executive function lower-level neurocognitive functions	I30–45 min per session, 4 times per week, 14 weeks;
de Bruijn et al., (2021)	fMRI	whole brain	Aerobic exercise Cognitively exercises Control group: regular exercise.	RCT	working memory	30–45 min per session, 4 times per week, 14 weeks
Tervaniemi et al., (2021)	EEG	whole brain	Music course English course	RCT	auditory function	30–45 min per session, 4 times per week, 14 weeks
Wang et al., (2019)	MRI fMRI	whole brain	AMC CG: normal school course.	RCT	visuospatial working memory	1 h/session, 2 times/ week, 50 sessions
Farah et al., (2019)	EEG	whole brain	DRG SSG	RCT	Narrative comprehension ability	30 min/session, 3 times/week, 6 weeks
Chobert et al., (2014)	EEG	whole brain	Music training Painting training	RCT	preattentive of syllabic duration and voice onset time	45 min/session, twice a week in 1st semster, once a week in 2nd semester, 6 months/year, 2 school years
Bauer et al., (2019)	fMRI	amygdala	Mindfulness training Coding training	RCT	stress regulation	45-min per session, 4 times per week, 8 weeks
Dettweiler et al., (2022)	fMRI MRI	Amygdala hippocampus ACC	IG: out door classes CG: indoor classes.	RCT	stress regulation	(5 +1) 45 min/day, 1 day/week, 1 school year
Zhang et al., (2024)	fNIRS	IFG, STG, AG	Magnetism, Speed, Friction	Non-RCT	/	/
Barreto et al., (2021)	fNIRS	PFC TPJ	the mechanisms of summing two numbers in 1–12	Non-RCT	/	5 years

*Note*. IG=Intervention group; CG=Control group; DRG=dialogic reading group; SSG=screen story group; AMC=Abacus-based mental calculatio; EEG=electroencephalogram; fMRI=functional magnetic resonance imaging; fNIRS=Functional near-infrared imaging; MEG=magnetoencephalography; PFC=prefrontal cortex; ACC=anterior cingulate cortex; IFG=inferior frontal gyrus; STG=superior temporal gyrus; AG=angular gyrus; TPJ=temporoparietal junction; UNI=Unidirectional; DIA=Dialogue; COL=Collaborative; STU=Student-led.

**Table:** 

**Authors/year**	**Result**				
	**Significant brain results (BR)**		**BR associate with other result**		
Xie et al., (2024)	1. DCCS: DLPFC, R-PSFC, MPFC in IG 2. GO/NO-GO: DLPFC, R-PSFC in IG; R-PSFC, R-TC in CG. 3. WM task: DLPFC, PSFC, MPFC in IG; R-DLPFC, R-TC in CG.		/		
Ding et al., (2024)	1. Post-test: BA10, BA46 under neutral and word conditions. 2. Post-test: BA10, BA46 in IG lower than that CG.		1. Word condition (ST2) of pre-test: L-MFG activation was negatively correlated with accuracy and RT. 2.Post-test ST2: RT showed a negative correlation with R-SFG.		
Zhong et al., (2024b)	EG: higher activation in the L-DLPFC during the post test of the WM task.		1.Negative correlation between RT and the difference in pre - and post brain activation. 2. Accuracy was positively correlated with the difference in pre - and post brain activation.		
Yeatman et al., (2024)	1. Visual cortex (letter/word regions, a): increased functional activation and structural changes. 2. Visual cortex (category-selective regions, b): enhanced category selectivity and adaptive reorganization. 3. Language-related regions: indirect functional modulation via visual-language network.		/		
Zhong et al., (2024a)	Higher activation in the R-DLPFC during the flanker task.		Significant positive correlation between improvement in RT or ACC and R-DLPFC activation.		
Mora-Gonzalez et al., (2023)	1. WM: Significantly increased, especially during the retention of the second stimulus in the temporal and frontal lobes, the retention of the third stimulus in the frontal lobe, and the retention of the temporal and occipital lobes in the fourth stimulus. 2. IC: the exercise group showed lower changes in current density in the forehead.		/		
Cheng et al., (2023)		Flanker task, the activation of IG’s R-DLPFC was higher in the post test than in the pre-test.			/
Meijer et al., (2022)		No any effect was found in study.			/
de Bruijn et al., (2021)		No significant change in the brain activity.			/
Tervaniemi et al., (2021)		1. Except for intensity anomalies, all abnormal and novel sounds can induce MMN and/or P3a. 2. Music group: had a larger MMN in the recording after training (time & group). 3. The P3a of the English group is larger than that of the music group.			/
Wang et al., (2019)		The activation level in the frontal, parietal, and occipital regions of the AMC group was higher than that of the control group.			The activation of the right middle frontal gyrus mediated the association between the arithmetic and VSWM of the AMC group.
Farah et al., (2019)		1. Story listening task: DRG (dialogic reading grouop) showed an increase in vocabulary and a decrease in functional connectivity 2. After intervention, the network strength and transitivity of DRG were significantly reduced, and the network efficiency was improved.			Greater network strength and transitivity are associated with an increase in vocabulary.
Chobert et al., (2014)		After training in music group, it was found that MMN showed lager amplitudes.			/
Bauer et al., (2019)		1.After intervention, the mindfulness group (MG) showed greater activation of the right amygdala when facing fearful faces compared to the coding group. 2. Mindfulness training leads to relatively strong functional connections between the right amygdala and the ventromedial PFC.			1. Positive correlation between the decrease in stress scores in MG and decrease in amygdala activation on fearful faces. 2. After intervention: Significant decrease in the activation of the amygdala on happy faces between the two groups.
Dettweiler et al., (2022)		1. Hippocampus: Structural changes mainly driven by age, with no significant additional effect of the intervention. 2. ACC: Perceived autonomy support promoted ACC maturation, with stronger effects in the outdoor education intervention.		1.Cortisol levels positively correlated with amygdala activation, indicating stress regulation effects in the intervention group.	
Zhang et al., (2024)		The level of brain synchronization in the upper left margin was positively correlated with STEM learning outcomes under story-based conditions.		1. IFG synchronization was positively correlated with STEM performance under the condition of storyboard. 2. The level of brain synchronicity under traditional teaching conditions is significantly lower than under other conditions.	
Barreto et al., (2021)		Vector regression is more suitable for channel analysis of hyperscanning than ordinary least square, and teachers’ cortical activity can be used to predict students’ cerebral hemodynamic responses (PFC & TPJ).		/	

*Note*. Mage=mean age; IG=Intervention group; CG=Control group; WM=working memory; DLPFC=dorsolateral prefrontal cortex; MPFC=medial prefrontal cortex; PSFC=posterior superior frontal cortex; TC=temporal cortex; BA=Brodmann area; MFG=middle frontal gyrus; SFG=superior frontal gyrus; ADHD=attention deficit hyperactivity disorder; ASD=autism; MMN=mismatch negative; VSWM=visuospatial working memory; IFG=inferior temporal gyrus; WM=working memory; IC=inhibitory control; ACC=anterior cingulated cortex; RT=reaction time; R-=right-, L-=left-; ↔=none effect; ↑=stronger activation; ↓=lower activation.

## Data Availability

Data will be made available on request.
